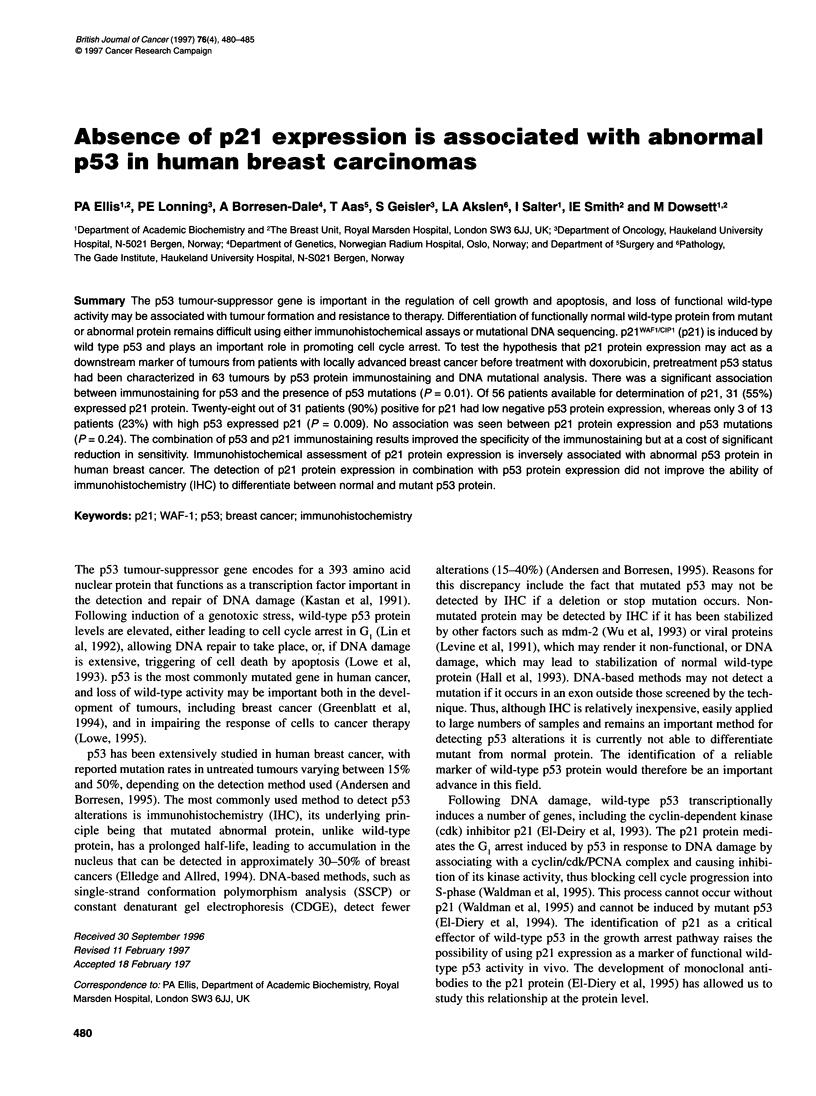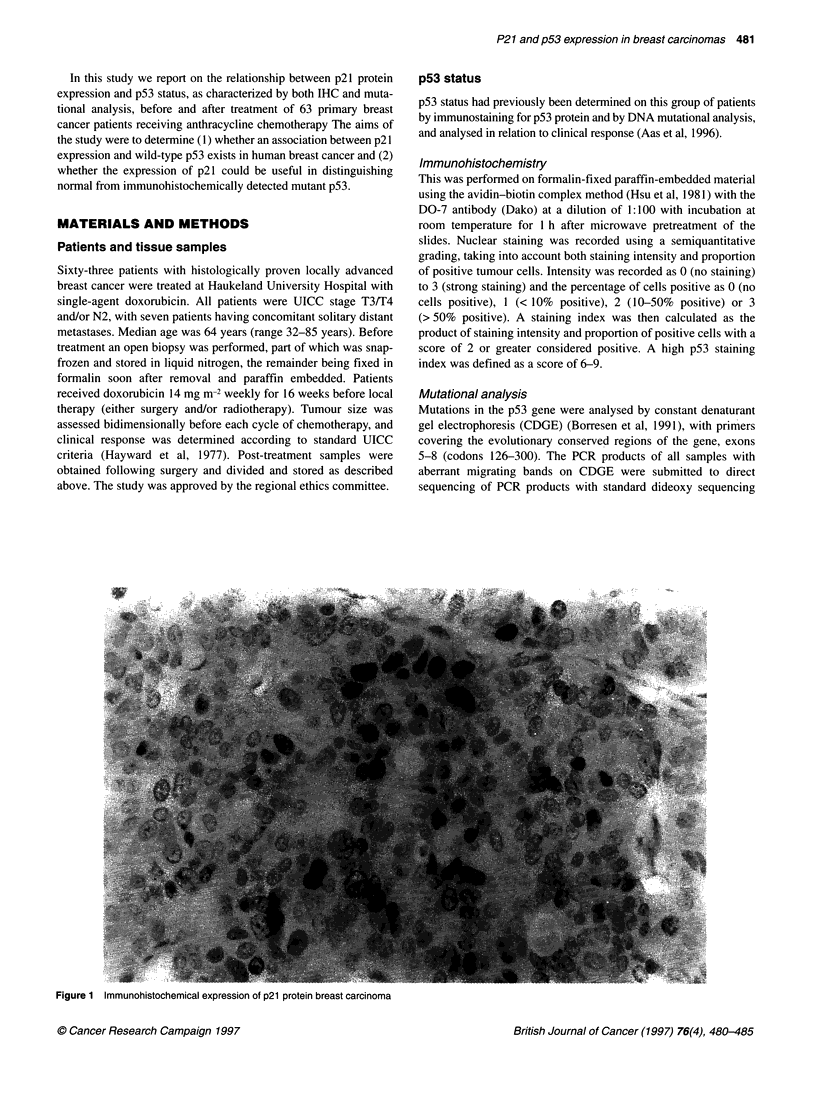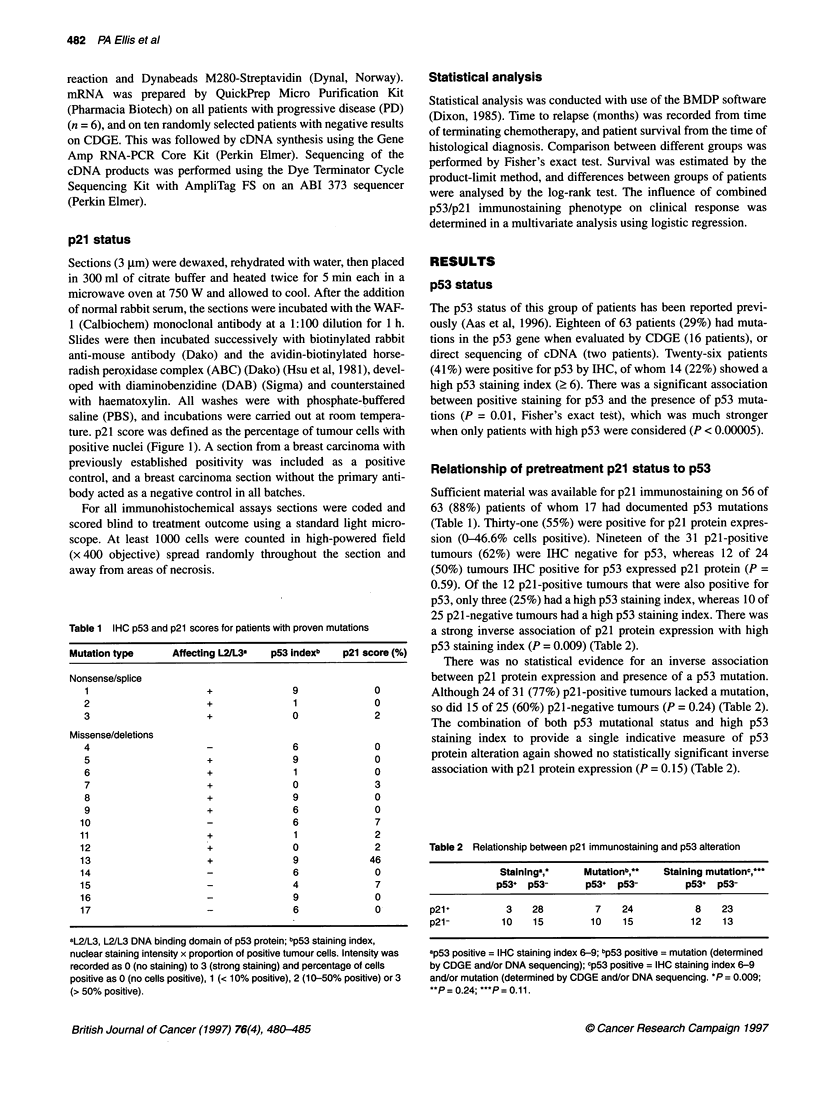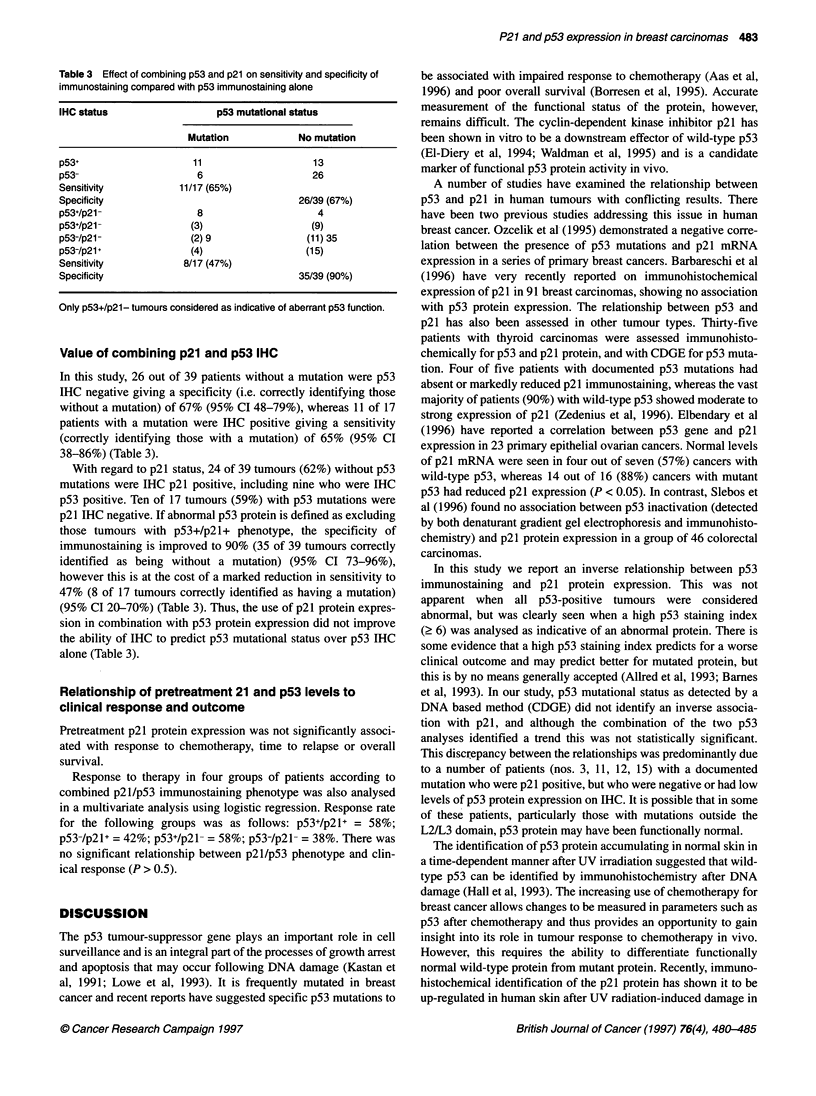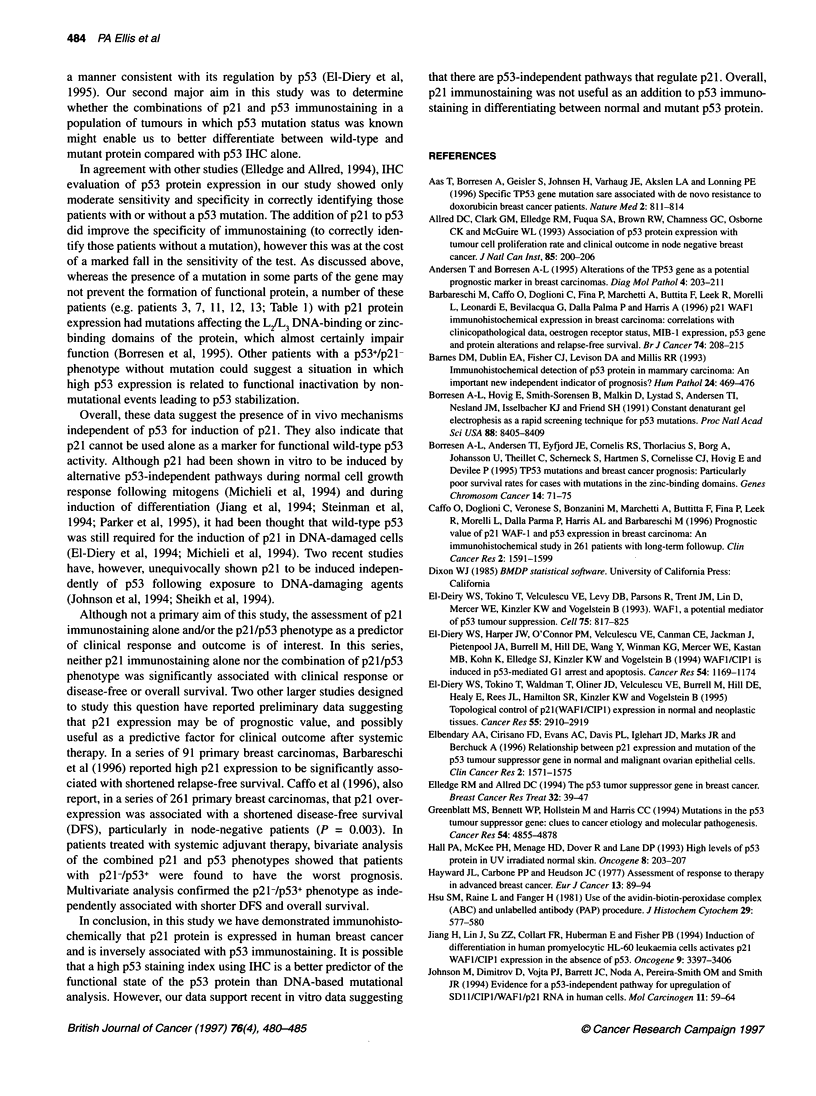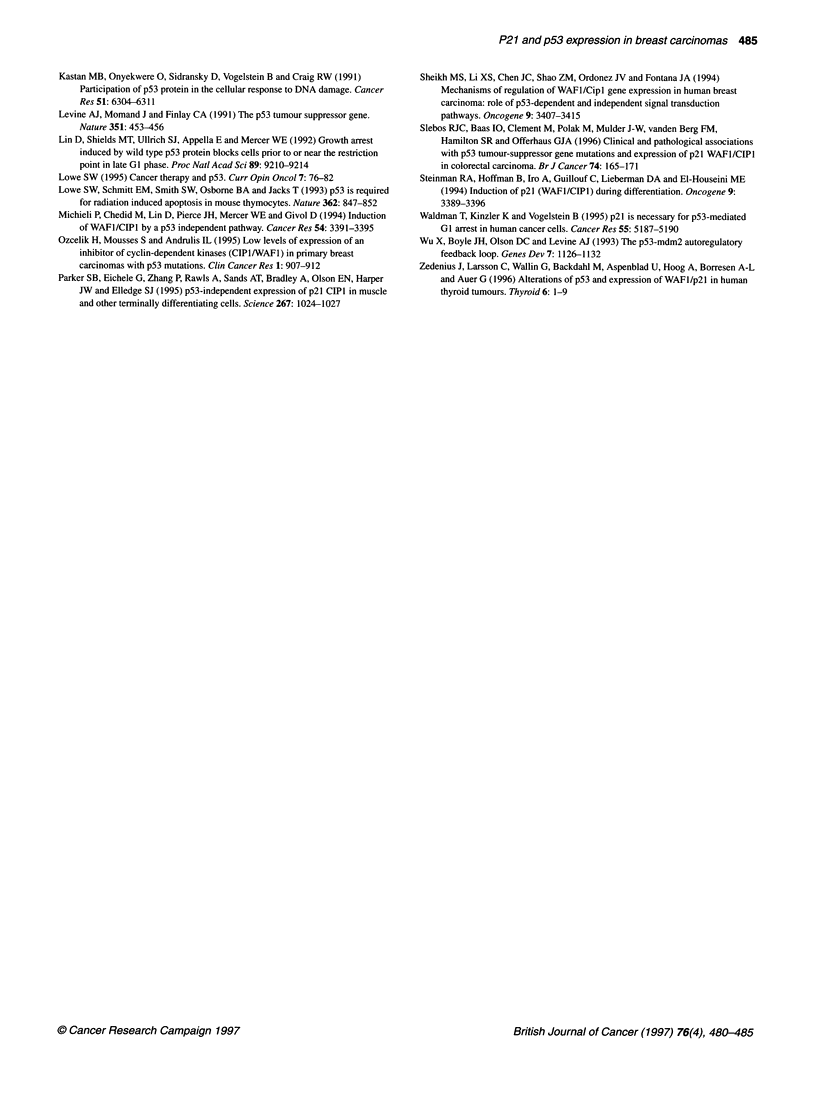# Absence of p21 expression is associated with abnormal p53 in human breast carcinomas.

**DOI:** 10.1038/bjc.1997.413

**Published:** 1997

**Authors:** P. A. Ellis, P. E. Lonning, A. Borresen-Dale, T. Aas, S. Geisler, L. A. Akslen, I. Salter, I. E. Smith, M. Dowsett

**Affiliations:** Department of Academic Biochemistry, Royal Marsden Hospital, London, UK.

## Abstract

**Images:**


*British Journal of Cancer* (1997) **76**(4), 480–485
© 1997 Cancer Research Campaign

### Absence of p21 expression is associated with abnormal p53 in human breast carcinomas

**PA Ellis[1,2], PE Lonning[3], A Borresen-Dale[4], T Aas[5], S Geisler[3], LA Akslen[6], I Salter[1], IE Smith[2] and M Dowsett[1,2]**

[1]Department of Academic Biochemistry and [2]The Breast Unit, Royal Marsden Hospital, London SW3 6JJ, UK; [3]Department of Oncology, Haukeland University Hospital, N-5021 Bergen, Norway; [4]Department of Genetics, Norwegian Radium Hospital, Oslo, Norway; and Department of [5]Surgery and [6]Pathology, The Gade Institute, Haukeland University Hospital, N-S021 Bergen, Norway

**Summary** The p53 tumour-suppressor gene is important in the regulation of cell growth and apoptosis, and loss of functional wild-type activity may be associated with tumour formation and resistance to therapy. Differentiation of functionally normal wild-type protein from mutant or abnormal protein remains difficult using either immunohistochemical assays or mutational DNA sequencing. $p21^{WAF1/CIP1}$ (p21) is induced by wild type p53 and plays an important role in promoting cell cycle arrest. To test the hypothesis that p21 protein expression may act as a downstream marker of tumours from patients with locally advanced breast cancer before treatment with doxorubicin, pretreatment p53 status had been characterized in 63 tumours by p53 protein immunostaining and DNA mutational analysis. There was a significant association between immunostaining for p53 and the presence of p53 mutations ($P = 0.01$). Of 56 patients available for determination of p21, 31 (55%) expressed p21 protein. Twenty-eight out of 31 patients (90%) positive for p21 had low negative p53 protein expression, whereas only 3 of 13 patients (23%) with high p53 expressed p21 ($P = 0.009$). No association was seen between p21 protein expression and p53 mutations ($P = 0.24$). The combination of p53 and p21 immunostaining results improved the specificity of the immunostaining but at a cost of significant reduction in sensitivity. Immunohistochemical assessment of p21 protein expression is inversely associated with abnormal p53 protein in human breast cancer. The detection of p21 protein expression in combination with p53 protein expression did not improve the ability of immunohistochemistry (IHC) to differentiate between normal and mutant p53 protein.

**Keywords:** p21; WAF-1; p53; breast cancer; immunohistochemistry

The p53 tumour-suppressor gene encodes for a 393 amino acid nuclear protein that functions as a transcription factor important in the detection and repair of DNA damage (Kastan et al, 1991). Following induction of a genotoxic stress, wild-type p53 protein levels are elevated, either leading to cell cycle arrest in $G_1$ (Lin et al, 1992), allowing DNA repair to take place, or, if DNA damage is extensive, triggering of cell death by apoptosis (Lowe et al, 1993). p53 is the most commonly mutated gene in human cancer, and loss of wild-type activity may be important both in the development of tumours, including breast cancer (Greenblatt et al, 1994), and in impairing the response of cells to cancer therapy (Lowe, 1995).

p53 has been extensively studied in human breast cancer, with reported mutation rates in untreated tumours varying between 15% and 50%, depending on the detection method used (Andersen and Borresen, 1995). The most commonly used method to detect p53 alterations is immunohistochemistry (IHC), its underlying principle being that mutated abnormal protein, unlike wild-type protein, has a prolonged half-life, leading to accumulation in the nucleus that can be detected in approximately 30–50% of breast cancers (Elledge and Allred, 1994). DNA-based methods, such as single-strand conformation polymorphism analysis (SSCP) or constant denaturant gel electrophoresis (CDGE), detect fewer alterations (15–40%) (Andersen and Borresen, 1995). Reasons for this discrepancy include the fact that mutated p53 may not be detected by IHC if a deletion or stop mutation occurs. Non-mutated protein may be detected by IHC if it has been stabilized by other factors such as mdm-2 (Wu et al, 1993) or viral proteins (Levine et al, 1991), which may render it non-functional, or DNA damage, which may lead to stabilization of normal wild-type protein (Hall et al, 1993). DNA-based methods may not detect a mutation if it occurs in an exon outside those screened by the technique. Thus, although IHC is relatively inexpensive, easily applied to large numbers of samples and remains an important method for detecting p53 alterations it is currently not able to differentiate mutant from normal protein. The identification of a reliable marker of wild-type p53 protein would therefore be an important advance in this field.

Following DNA damage, wild-type p53 transcriptionally induces a number of genes, including the cyclin-dependent kinase (cdk) inhibitor p21 (El-Deiry et al, 1993). The p21 protein mediates the $G_1$ arrest induced by p53 in response to DNA damage by associating with a cyclin/cdk/PCNA complex and causing inhibition of its kinase activity, thus blocking cell cycle progression into S-phase (Waldman et al, 1995). This process cannot occur without p21 (Waldman et al, 1995) and cannot be induced by mutant p53 (El-Diery et al, 1994). The identification of p21 as a critical effector of wild-type p53 in the growth arrest pathway raises the possibility of using p21 expression as a marker of functional wild-type p53 activity in vivo. The development of monoclonal antibodies to the p21 protein (El-Diery et al, 1995) has allowed us to study this relationship at the protein level.

*Received 30 September 1996*
*Revised 11 February 1997*
*Accepted 18 February 197*

*Correspondence to:* PA Ellis, Department of Academic Biochemistry, Royal Marsden Hospital, London SW3 6JJ, UK

In this study we report on the relationship between p21 protein expression and p53 status, as characterized by both IHC and mutational analysis, before and after treatment of 63 primary breast cancer patients receiving anthracycline chemotherapy The aims of the study were to determine (1) whether an association between p21 expression and wild-type p53 exists in human breast cancer and (2) whether the expression of p21 could be useful in distinguishing normal from immunohistochemically detected mutant p53.

### MATERIALS AND METHODS

### Patients and tissue samples

Sixty-three patients with histologically proven locally advanced breast cancer were treated at Haukeland University Hospital with single-agent doxorubicin. All patients were UICC stage T3/T4 and/or N2, with seven patients having concomitant solitary distant metastases. Median age was 64 years (range 32–85 years). Before treatment an open biopsy was performed, part of which was snap-frozen and stored in liquid nitrogen, the remainder being fixed in formalin soon after removal and paraffin embedded. Patients received doxorubicin 14 mg $m^{-2}$ weekly for 16 weeks before local therapy (either surgery and/or radiotherapy). Tumour size was assessed bidimensionally before each cycle of chemotherapy, and clinical response was determined according to standard UICC criteria (Hayward et al, 1977). Post-treatment samples were obtained following surgery and divided and stored as described above. The study was approved by the regional ethics committee.

### p53 status p53 status had previously been determined on this group of patients by immunostaining for p53 protein and by DNA mutational analysis, and analysed in relation to clinical response (Aas et al, 1996).

#### *Immunohistochemistry*

This was performed on formalin-fixed paraffin-embedded material using the avidin–biotin complex method (Hsu et al, 1981) with the DO-7 antibody (Dako) at a dilution of 1:100 with incubation at room temperature for 1 h after microwave pretreatment of the slides. Nuclear staining was recorded using a semiquantitative grading, taking into account both staining intensity and proportion of positive tumour cells. Intensity was recorded as 0 (no staining) to 3 (strong staining) and the percentage of cells positive as 0 (no cells positive), 1 (< 10% positive), 2 (10–50% positive) or 3 (> 50% positive). A staining index was then calculated as the product of staining intensity and proportion of positive cells with a score of 2 or greater considered positive. A high p53 staining index was defined as a score of 6–9.

#### *Mutational analysis*

Mutations in the p53 gene were analysed by constant denaturant gel electrophoresis (CDGE) (Borresen et al, 1991), with primers covering the evolutionary conserved regions of the gene, exons 5–8 (codons 126–300). The PCR products of all samples with aberrant migrating bands on CDGE were submitted to direct sequencing of PCR products with standard dideoxy sequencing

**Figure 1** Immunohistochemical expression of p21 protein breast carcinoma

© Cancer Research Campaign 1997

reaction and Dynabeads M280-Streptavidin (Dynal, Norway). mRNA was prepared by QuickPrep Micro Purification Kit (Pharmacia Biotech) on all patients with progressive disease (PD) ($n = 6$), and on ten randomly selected patients with negative results on CDGE. This was followed by cDNA synthesis using the Gene Amp RNA-PCR Core Kit (Perkin Elmer). Sequencing of the cDNA products was performed using the Dye Terminator Cycle Sequencing Kit with AmpliTag FS on an ABI 373 sequencer (Perkin Elmer).

### p21 status

Sections (3 μm) were dewaxed, rehydrated with water, then placed in 300 ml of citrate buffer and heated twice for 5 min each in a microwave oven at 750 W and allowed to cool. After the addition of normal rabbit serum, the sections were incubated with the WAF-1 (Calbiochem) monoclonal antibody at a 1:100 dilution for 1 h. Slides were then incubated successively with biotinylated rabbit anti-mouse antibody (Dako) and the avidin-biotinylated horseradish peroxidase complex (ABC) (Dako) (Hsu et al, 1981), developed with diaminobenzidine (DAB) (Sigma) and counterstained with haematoxylin. All washes were with phosphate-buffered saline (PBS), and incubations were carried out at room temperature. p21 score was defined as the percentage of tumour cells with positive nuclei (Figure 1). A section from a breast carcinoma with previously established positivity was included as a positive control, and a breast carcinoma section without the primary antibody acted as a negative control in all batches.

For all immunohistochemical assays sections were coded and scored blind to treatment outcome using a standard light microscope. At least 1000 cells were counted in high-powered field (× 400 objective) spread randomly throughout the section and away from areas of necrosis.

**Table 1** IHC p53 and p21 scores for patients with proven mutations

| Mutation type | Affecting L2/L3[a] | p53 index[b] | p21 score (%) |
|---|---|---|---|
| Nonsense/splice | | | |
| 1 | + | 9 | 0 |
| 2 | + | 1 | 0 |
| 3 | + | 0 | 2 |
| Missense/deletions | | | |
| 4 | – | 6 | 0 |
| 5 | + | 9 | 0 |
| 6 | + | 1 | 0 |
| 7 | + | 0 | 3 |
| 8 | + | 9 | 0 |
| 9 | + | 6 | 0 |
| 10 | – | 6 | 7 |
| 11 | + | 1 | 2 |
| 12 | + | 0 | 2 |
| 13 | + | 9 | 46 |
| 14 | – | 6 | 0 |
| 15 | – | 4 | 7 |
| 16 | – | 9 | 0 |
| 17 | – | 6 | 0 |

[a]L2/L3, L2/L3 DNA binding domain of p53 protein; [b]p53 staining index, nuclear staining intensity × proportion of positive tumour cells. Intensity was recorded as 0 (no staining) to 3 (strong staining) and percentage of cells positive as 0 (no cells positive), 1 (< 10% positive), 2 (10–50% positive) or 3 (> 50% positive).

### Statistical analysis

Statistical analysis was conducted with use of the BMDP software (Dixon, 1985). Time to relapse (months) was recorded from time of terminating chemotherapy, and patient survival from the time of histological diagnosis. Comparison between different groups was performed by Fisher's exact test. Survival was estimated by the product-limit method, and differences between groups of patients were analysed by the log-rank test. The influence of combined p53/p21 immunostaining phenotype on clinical response was determined in a multivariate analysis using logistic regression.

### RESULTS

### p53 status

The p53 status of this group of patients has been reported previously (Aas et al, 1996). Eighteen of 63 patients (29%) had mutations in the p53 gene when evaluated by CDGE (16 patients), or direct sequencing of cDNA (two patients). Twenty-six patients (41%) were positive for p53 by IHC, of whom 14 (22%) showed a high p53 staining index (≥ 6). There was a significant association between positive staining for p53 and the presence of p53 mutations ($P = 0.01$, Fisher's exact test), which was much stronger when only patients with high p53 were considered ($P < 0.00005$).

### Relationship of pretreatment p21 status to p53

Sufficient material was available for p21 immunostaining on 56 of 63 (88%) patients of whom 17 had documented p53 mutations (Table 1). Thirty-one (55%) were positive for p21 protein expression (0–46.6% cells positive). Nineteen of the 31 p21-positive tumours (62%) were IHC negative for p53, whereas 12 of 24 (50%) tumours IHC positive for p53 expressed p21 protein ($P = 0.59$). Of the 12 p21-positive tumours that were also positive for p53, only three (25%) had a high p53 staining index, whereas 10 of 25 p21-negative tumours had a high p53 staining index. There was a strong inverse association of p21 protein expression with high p53 staining index ($P = 0.009$) (Table 2).

There was no statistical evidence for an inverse association between p21 protein expression and presence of a p53 mutation. Although 24 of 31 (77%) p21-positive tumours lacked a mutation, so did 15 of 25 (60%) p21-negative tumours ($P = 0.24$) (Table 2). The combination of both p53 mutational status and high p53 staining index to provide a single indicative measure of p53 protein alteration again showed no statistically significant inverse association with p21 protein expression ($P = 0.15$) (Table 2).

**Table 2** Relationship between p21 immunostaining and p53 alteration

| | Staining[a],* | | Mutation[b],** | | Staining mutation[c],*** | |
|---|---|---|---|---|---|---|
| | $p53^+$ | $p53^-$ | $p53^+$ | $p53^-$ | $p53^+$ | $p53^-$ |
| $p21^+$ | 3 | 28 | 7 | 24 | 8 | 23 |
| $p21^-$ | 10 | 15 | 10 | 15 | 12 | 13 |

[a]p53 positive = IHC staining index 6–9; [b]p53 positive = mutation (determined by CDGE and/or DNA sequencing); [c]p53 positive = IHC staining index 6–9 and/or mutation (determined by CDGE and/or DNA sequencing. *$P = 0.009$; **$P = 0.24$; ***$P = 0.11$.

© Cancer Research Campaign 1997

**Table 3** Effect of combining p53 and p21 on sensitivity and specificity of immunostaining compared with p53 immunostaining alone

| IHC status | p53 mutational status | |
|---|---|---|
| | Mutation | No mutation |
| $p53^+$ | 11 | 13 |
| $p53^-$ | 6 | 26 |
| Sensitivity | 11/17 (65%) | |
| Specificity | | 26/39 (67%) |
| $p53^+/p21^-$ | 8 | 4 |
| $p53^+/p21^-$ | (3) | (9) |
| $p53^-/p21^-$ | (2) 9 | (11) 35 |
| $p53^-/p21^+$ | (4) | (15) |
| Sensitivity | 8/17 (47%) | |
| Specificity | | 35/39 (90%) |

Only p53+/p21– tumours considered as indicative of aberrant p53 function.

### Value of combining p21 and p53 IHC

In this study, 26 out of 39 patients without a mutation were p53 IHC negative giving a specificity (i.e. correctly identifying those without a mutation) of 67% (95% CI 48–79%), whereas 11 of 17 patients with a mutation were IHC positive giving a sensitivity (correctly identifying those with a mutation) of 65% (95% CI 38–86%) (Table 3).

With regard to p21 status, 24 of 39 tumours (62%) without p53 mutations were IHC p21 positive, including nine who were IHC p53 positive. Ten of 17 tumours (59%) with p53 mutations were p21 IHC negative. If abnormal p53 protein is defined as excluding those tumours with p53+/p21+ phenotype, the specificity of immunostaining is improved to 90% (35 of 39 tumours correctly identified as being without a mutation) (95% CI 73–96%), however this is at the cost of a marked reduction in sensitivity to 47% (8 of 17 tumours correctly identified as having a mutation) (95% CI 20–70%) (Table 3). Thus, the use of p21 protein expression in combination with p53 protein expression did not improve the ability of IHC to predict p53 mutational status over p53 IHC alone (Table 3).

### Relationship of pretreatment 21 and p53 levels to clinical response and outcome

Pretreatment p21 protein expression was not significantly associated with response to chemotherapy, time to relapse or overall survival.

Response to therapy in four groups of patients according to combined p21/p53 immunostaining phenotype was also analysed in a multivariate analysis using logistic regression. Response rate for the following groups was as follows: $p53^+/p21^+$ = 58%; $p53^-/p21^+$ = 42%; $p53^+/p21^-$ = 58%; $p53^-/p21^-$ = 38%. There was no significant relationship between p21/p53 phenotype and clinical response ($P > 0.5$).

### DISCUSSION

The p53 tumour-suppressor gene plays an important role in cell surveillance and is an integral part of the processes of growth arrest and apoptosis that may occur following DNA damage (Kastan et al, 1991; Lowe et al, 1993). It is frequently mutated in breast cancer and recent reports have suggested specific p53 mutations to be associated with impaired response to chemotherapy (Aas et al, 1996) and poor overall survival (Borresen et al, 1995). Accurate measurement of the functional status of the protein, however, remains difficult. The cyclin-dependent kinase inhibitor p21 has been shown in vitro to be a downstream effector of wild-type p53 (El-Diery et al, 1994; Waldman et al, 1995) and is a candidate marker of functional p53 protein activity in vivo.

A number of studies have examined the relationship between p53 and p21 in human tumours with conflicting results. There have been two previous studies addressing this issue in human breast cancer. Ozcelik et al (1995) demonstrated a negative correlation between the presence of p53 mutations and p21 mRNA expression in a series of primary breast cancers. Barbareschi et al (1996) have very recently reported on immunohistochemical expression of p21 in 91 breast carcinomas, showing no association with p53 protein expression. The relationship between p53 and p21 has also been assessed in other tumour types. Thirty-five patients with thyroid carcinomas were assessed immunohistochemically for p53 and p21 protein, and with CDGE for p53 mutation. Four of five patients with documented p53 mutations had absent or markedly reduced p21 immunostaining, whereas the vast majority of patients (90%) with wild-type p53 showed moderate to strong expression of p21 (Zedenius et al, 1996). Elbendary et al (1996) have reported a correlation between p53 gene and p21 expression in 23 primary epithelial ovarian cancers. Normal levels of p21 mRNA were seen in four out of seven (57%) cancers with wild-type p53, whereas 14 out of 16 (88%) cancers with mutant p53 had reduced p21 expression ($P < 0.05$). In contrast, Slebos et al (1996) found no association between p53 inactivation (detected by both denaturant gradient gel electrophoresis and immunohistochemistry) and p21 protein expression in a group of 46 colorectal carcinomas.

In this study we report an inverse relationship between p53 immunostaining and p21 protein expression. This was not apparent when all p53-positive tumours were considered abnormal, but was clearly seen when a high p53 staining index ($\geq 6$) was analysed as indicative of an abnormal protein. There is some evidence that a high p53 staining index predicts for a worse clinical outcome and may predict better for mutated protein, but this is by no means generally accepted (Allred et al, 1993; Barnes et al, 1993). In our study, p53 mutational status as detected by a DNA based method (CDGE) did not identify an inverse association with p21, and although the combination of the two p53 analyses identified a trend this was not statistically significant. This discrepancy between the relationships was predominantly due to a number of patients (nos. 3, 11, 12, 15) with a documented mutation who were p21 positive, but who were negative or had low levels of p53 protein expression on IHC. It is possible that in some of these patients, particularly those with mutations outside the L2/L3 domain, p53 protein may have been functionally normal.

The identification of p53 protein accumulating in normal skin in a time-dependent manner after UV irradiation suggested that wild-type p53 can be identified by immunohistochemistry after DNA damage (Hall et al, 1993). The increasing use of chemotherapy for breast cancer allows changes to be measured in parameters such as p53 after chemotherapy and thus provides an opportunity to gain insight into its role in tumour response to chemotherapy in vivo. However, this requires the ability to differentiate functionally normal wild-type protein from mutant protein. Recently, immunohistochemical identification of the p21 protein has shown it to be up-regulated in human skin after UV radiation-induced damage in

© Cancer Research Campaign 1997 

a manner consistent with its regulation by p53 (El-Diery et al, 1995). Our second major aim in this study was to determine whether the combinations of p21 and p53 immunostaining in a population of tumours in which p53 mutation status was known might enable us to better differentiate between wild-type and mutant protein compared with p53 IHC alone.

In agreement with other studies (Elledge and Allred, 1994), IHC evaluation of p53 protein expression in our study showed only moderate sensitivity and specificity in correctly identifying those patients with or without a p53 mutation. The addition of p21 to p53 did improve the specificity of immunostaining (to correctly identify those patients without a mutation), however this was at the cost of a marked fall in the sensitivity of the test. As discussed above, whereas the presence of a mutation in some parts of the gene may not prevent the formation of functional protein, a number of these patients (e.g. patients 3, 7, 11, 12, 13; Table 1) with p21 protein expression had mutations affecting the $L_2/L_3$ DNA-binding or zinc-binding domains of the protein, which almost certainly impair function (Borresen et al, 1995). Other patients with a $p53^+/p21^-$ phenotype without mutation could suggest a situation in which high p53 expression is related to functional inactivation by non-mutational events leading to p53 stabilization.

Overall, these data suggest the presence of in vivo mechanisms independent of p53 for induction of p21. They also indicate that p21 cannot be used alone as a marker for functional wild-type p53 activity. Although p21 had been shown in vitro to be induced by alternative p53-independent pathways during normal cell growth response following mitogens (Michieli et al, 1994) and during induction of differentiation (Jiang et al, 1994; Steinman et al, 1994; Parker et al, 1995), it had been thought that wild-type p53 was still required for the induction of p21 in DNA-damaged cells (El-Diery et al, 1994; Michieli et al, 1994). Two recent studies have, however, unequivocally shown p21 to be induced independently of p53 following exposure to DNA-damaging agents (Johnson et al, 1994; Sheikh et al, 1994).

Although not a primary aim of this study, the assessment of p21 immunostaining alone and/or the p21/p53 phenotype as a predictor of clinical response and outcome is of interest. In this series, neither p21 immunostaining alone nor the combination of p21/p53 phenotype was significantly associated with clinical response or disease-free or overall survival. Two other larger studies designed to study this question have reported preliminary data suggesting that p21 expression may be of prognostic value, and possibly useful as a predictive factor for clinical outcome after systemic therapy. In a series of 91 primary breast carcinomas, Barbareschi et al (1996) reported high p21 expression to be significantly associated with shortened relapse-free survival. Caffo et al (1996), also report, in a series of 261 primary breast carcinomas, that p21 overexpression was associated with a shortened disease-free survival (DFS), particularly in node-negative patients ($P$ = 0.003). In patients treated with systemic adjuvant therapy, bivariate analysis of the combined p21 and p53 phenotypes showed that patients with $p21^-/p53^+$ were found to have the worst prognosis. Multivariate analysis confirmed the $p21^-/p53^+$ phenotype as independently associated with shorter DFS and overall survival.

In conclusion, in this study we have demonstrated immunohistochemically that p21 protein is expressed in human breast cancer and is inversely associated with p53 immunostaining. It is possible that a high p53 staining index using IHC is a better predictor of the functional state of the p53 protein than DNA-based mutational analysis. However, our data support recent in vitro data suggesting that there are p53-independent pathways that regulate p21. Overall, p21 immunostaining was not useful as an addition to p53 immunostaining in differentiating between normal and mutant p53 protein.

### REFERENCES

Aas T, Borresen A, Geisler S, Johnsen H, Varhaug JE, Akslen LA and Lonning PE (1996) Specific TP53 gene mutation sare associated with de novo resistance to doxorubicin breast cancer patients. *Nature Med* **2**: 811–814

Allred DC, Clark GM, Elledge RM, Fuqua SA, Brown RW, Chamness GC, Osborne CK and McGuire WL (1993) Association of p53 protein expression with tumour cell proliferation rate and clinical outcome in node negative breast cancer. *J Natl Can Inst*, **85**: 200–206

Andersen T and Borresen A-L (1995) Alterations of the TP53 gene as a potential prognostic marker in breast carcinomas. *Diag Mol Pathol* **4**: 203–211

Barbareschi M, Caffo O, Doglioni C, Fina P, Marchetti A, Buttita F, Leek R, Morelli L, Leonardi E, Bevilacqua G, Dalla Palma P and Harris A (1996) p21 WAF1 immunohistochemical expression in breast carcinoma: correlations with clinicopathological data, oestrogen receptor status, MIB-1 expression, p53 gene and protein alterations and relapse-free survival. *Br J Cancer* **74**: 208–215

Barnes DM, Dublin EA, Fisher CJ, Levison DA and Millis RR (1993) Immunohistochemical detection of p53 protein in mammary carcinoma: An important new independent indicator of prognosis? *Hum Pathol* **24**: 469–476

Borresen A-L, Hovig E, Smith-Sorensen B, Malkin D, Lystad S, Andersen TI, Nesland JM, Isselbacher KJ and Friend SH (1991) Constant denaturant gel electrophesis as a rapid screening technique for p53 mutations. *Proc Natl Acad Sci USA* **88**: 8405–8409

Borresen A-L, Andersen TI, Eyfjord JE, Cornelis RS, Thorlacius S, Borg A, Johansson U, Theillet C, Scherneck S, Hartmen S, Cornelisse CJ, Hovig E and Devilee P (1995) TP53 mutations and breast cancer prognosis: Particularly poor survival rates for cases with mutations in the zinc-binding domains. *Genes Chromosom Cancer* **14**: 71–75

Caffo O, Doglioni C, Veronese S, Bonzanini M, Marchetti A, Buttitta F, Fina P, Leek R, Morelli L, Dalla Parma P, Harris AL and Barbareschi M (1996) Prognostic value of p21 WAF-1 and p53 expression in breast carcinoma: An immunohistochemical study in 261 patients with long-term followup. *Clin Cancer Res* **2**: 1591–1599

Dixon WJ (1985) *BMDP statistical software*. University of California Press: California

El-Deiry WS, Tokino T, Velculescu VE, Levy DB, Parsons R, Trent JM, Lin D, Mercer WE, Kinzler KW and Vogelstein B (1993). WAF1, a potential mediator of p53 tumour suppression. *Cell* **75**: 817–825

El-Diery WS, Harper JW, O'Connor PM, Velculescu VE, Canman CE, Jackman J, Pietenpool JA, Burrell M, Hill DE, Wang Y, Winman KG, Mercer WE, Kastan MB, Kohn K, Elledge SJ, Kinzler KW and Vogelstein B (1994) WAF1/CIP1 is induced in p53-mediated G1 arrest and apoptosis. *Cancer Res* **54**: 1169–1174

El-Diery WS, Tokino T, Waldman T, Oliner JD, Velculescu VE, Burrell M, Hill DE, Healy E, Rees JL, Hamilton SR, Kinzler KW and Vogelstein B (1995) Topological control of p21(WAF1/CIP1) expression in normal and neoplastic tissues. *Cancer Res* **55**: 2910–2919

Elbendary AA, Cirisano FD, Evans AC, Davis PL, Iglehart JD, Marks JR and Berchuck A (1996) Relationship between p21 expression and mutation of the p53 tumour suppressor gene in normal and malignant ovarian epithelial cells. *Clin Cancer Res* **2**: 1571–1575

Elledge RM and Allred DC (1994) The p53 tumor suppressor gene in breast cancer. *Breast Cancer Res Treat* **32**: 39–47

Greenblatt MS, Bennett WP, Hollstein M and Harris CC (1994) Mutations in the p53 tumour suppressor gene: clues to cancer etiology and molecular pathogenesis. *Cancer Res* **54**: 4855–4878

Hall PA, McKee PH, Menage HD, Dover R and Lane DP (1993) High levels of p53 protein in UV irradiated normal skin. *Oncogene* **8**: 203–207

Hayward JL, Carbone PP and Heudson JC (1977) Assessment of response to therapy in advanced breast cancer. *Eur J Cancer* **13**: 89–94

Hsu SM, Raine L and Fanger H (1981) Use of the avidin-biotin-peroxidase complex (ABC) and unlabelled antibody (PAP) procedure. *J Histochem Cytochem* **29**: 577–580

Jiang H, Lin J, Su ZZ, Collart FR, Huberman E and Fisher PB (1994) Induction of differentiation in human promyelocytic HL-60 leukaemia cells activates p21 WAF1/CIP1 expression in the absence of p53. *Oncogene* **9**: 3397–3406

Johnson M, Dimitrov D, Vojta PJ, Barrett JC, Noda A, Pereira-Smith OM and Smith JR (1994) Evidence for a p53-independent pathway for upregulation of SD11/CIP1/WAF1/p21 RNA in human cells. *Mol Carcinogen* **11**: 59–64

© Cancer Research Campaign 1997

Kastan MB, Onyekwere O, Sidransky D, Vogelstein B and Craig RW (1991) Participation of p53 protein in the cellular response to DNA damage. *Cancer Res* **51**: 6304–6311

Levine AJ, Momand J and Finlay CA (1991) The p53 tumour suppressor gene. *Nature* **351**: 453–456

Lin D, Shields MT, Ullrich SJ, Appella E and Mercer WE (1992) Growth arrest induced by wild type p53 protein blocks cells prior to or near the restriction point in late G1 phase. *Proc Natl Acad Sci* **89**: 9210–9214

Lowe SW (1995) Cancer therapy and p53. *Curr Opin Oncol* **7**: 76–82

Lowe SW, Schmitt EM, Smith SW, Osborne BA and Jacks T (1993) p53 is required for radiation induced apoptosis in mouse thymocytes. *Nature* **362**: 847–852

Michieli P, Chedid M, Lin D, Pierce JH, Mercer WE and Givol D (1994) Induction of WAF1/CIP1 by a p53 independent pathway. *Cancer Res* **54**: 3391–3395

Ozcelik H, Mousses S and Andrulis IL (1995) Low levels of expression of an inhibitor of cyclin-dependent kinases (CIP1/WAF1) in primary breast carcinomas with p53 mutations. *Clin Cancer Res* **1**: 907–912

Parker SB, Eichele G, Zhang P, Rawls A, Sands AT, Bradley A, Olson EN, Harper JW and Elledge SJ (1995) p53-independent expression of p21 CIP1 in muscle and other terminally differentiating cells. *Science* **267**: 1024–1027

Sheikh MS, Li XS, Chen JC, Shao ZM, Ordonez JV and Fontana JA (1994) Mechanisms of regulation of WAF1/Cip1 gene expression in human breast carcinoma: role of p53-dependent and independent signal transduction pathways. *Oncogene* **9**: 3407–3415

Slebos RJC, Baas IO, Clement M, Polak M, Mulder J-W, vanden Berg FM, Hamilton SR and Offerhaus GJA (1996) Clinical and pathological associations with p53 tumour-suppressor gene mutations and expression of p21 WAF1/CIP1 in colorectal carcinoma. *Br J Cancer* **74**: 165–171

Steinman RA, Hoffman B, Iro A, Guillouf C, Lieberman DA and El-Houseini ME (1994) Induction of p21 (WAF1/CIP1) during differentiation. *Oncogene* **9**: 3389–3396

Waldman T, Kinzler K and Vogelstein B (1995) p21 is necessary for p53-mediated G1 arrest in human cancer cells. *Cancer Res* **55**: 5187–5190

Wu X, Boyle JH, Olson DC and Levine AJ (1993) The p53-mdm2 autoregulatory feedback loop. *Genes Dev* **7**: 1126–1132

Zedenius J, Larsson C, Wallin G, Backdahl M, Aspenblad U, Hoog A, Borresen A-L and Auer G (1996) Alterations of p53 and expression of WAF1/p21 in human thyroid tumours. *Thyroid* **6**: 1–9

© Cancer Research Campaign 1997